# Possible limitations of dolphin echolocation: a simulation study based on a cross-modal matching experiment

**DOI:** 10.1038/s41598-021-85063-2

**Published:** 2021-03-23

**Authors:** Chong Wei, Matthias Hoffmann-Kuhnt, Whitlow W. L. Au, Abel Zhong Hao Ho, Eszter Matrai, Wen Feng, Darlene R. Ketten, Yu Zhang

**Affiliations:** 1 Centre for Marine Science and Technology, Curtin University, Kent Street, Bentley, WA 6102 Australia; 2 Acoustic Research Laboratory, Tropical Marine Science Institute, National University of Singapore, 18 Kent Ridge Road, Singapore, 119227 Singapore; 3 Hawaii Institute of Marine Biology, University of Hawaii, 46-007 Lilipuna Road, Kaneohe, HI 96744 USA; 4 Research Department, Ocean Park Hong Kong, Hong Kong (SAR), China; 5 School of Information Engineering, Jimei University, Xiamen, 361021 People’s Republic of China; 6 Department of Biomedical Engineering, Boston University, Boston, MA 02215 USA; 7 Department of Otology and Laryngology, Harvard Medical School, Biology Department, Woods Hole Oceanographic Institution, Woods Hole, MA USA; 8 Key Laboratory of Underwater Acoustic Communication and Marine Information Technology of the Ministry of Education, Xiamen University, Xiangan South Road, Xiamen, 361100 People’s Republic of China; 9 College of Oceanography and Environmental Science, Xiamen University, Xiangan South Road, Xiamen, 361100 People’s Republic of China

**Keywords:** Biological physics, Marine biology, Computational biophysics, Zoology

## Abstract

Dolphins use their biosonar to discriminate objects with different features through the returning echoes. Cross-modal matching experiments were conducted with a resident bottlenose dolphin (*Tursiops aduncus*). Four types of objects composed of different materials (water-filled PVC pipes, air-filled PVC pipes, foam ball arrays, and PVC pipes wrapped in closed-cell foam) were used in the experiments, respectively. The size and position of the objects remained the same in each case. The data collected in the experiment showed that the dolphin’s matching accuracy was significantly different across the cases. To gain insight into the underlying mechanism in the experiments, we used finite element methods to construct two-dimensional target detection models of an echolocating dolphin in the vertical plane, based on computed tomography scan data. The acoustic processes of the click’s interaction with the objects and the surrounding media in the four cases were simulated and compared. The simulation results provide some possible explanations for why the dolphin performed differently when discriminating the objects that only differed in material composition in the previous matching experiments.

## Introduction

Dolphins use their biological sonar (biosonar) system to detect objects of interest in their environment. Their ability to detect objects within a certain range is unique, even if or when the size of the objects is a couple of centimetres. For instance, Atlantic bottlenose dolphins (*Tursiops truncatus*) can detect a stainless-steel sphere of size 7.62 cm suspended in the water at distances further than 100 m^1^. They are also able to detect a 2.54 cm diameter solid steel sphere at a distance further than 70 m^2^. In addition to detection, dolphins can discriminate the features of objects, including structure, shape, material composition, and size by processing the returning echoes. Early experimental work showed that dolphins are able to discriminate disks differing in diameter by 0.9 cm at a distance of 0.7 m^3^ and aluminium cylinders differing in wall thickness by 0.23 mm at a distance of 8 m^4^. Dolphins can discriminate objects with various shapes such as cylinders, cubes, and spheres^5,6^. Their biosonar system can recognize the shapes of various objects based on the acoustic cues from the echoes, such as amplitude, spectral content, highlight structure, etc. To investigate how dolphins perceive object properties through echolocation, DeLong et al.^7^ conducted echoic-to-echoic (E–E) matching-to-sample (MTS) experiments to demonstrate that dolphins use multiple features and integrate information across the reflected echoes from a range of object orientations to identify the targets that varied in size, shape, material, and texture.

In addition to target detection and discrimination only by echolocation, Pack, Herman, and their colleagues^8–11^ conducted a series of cross-modal MTS experiments and presented a theory that dolphins can “see” through sound. They suggested that dolphins could perceive shape through echoic detection, and that the representation of the object echolocated on was then also accessible to their visual sense (echoic-to-visual matching-to-sample), then vice-versa. A variety of shapes built with polyvinyl chloride (PVC) pipes and fittings were used in the experiments. Several bottlenose dolphins were trained to perform echoic-visual (E-V) and visual-echoic (V-E) cross-modal MTS experiments. In the experiments, Pack and Herman’s group showed that dolphins were able to use their echolocation sense alone to inspect a complex shaped sample object, and subsequently find the match between several alternative objects through visual sense alone without any prior exposure to these objects. With a variety of sample shapes the dolphins performance was significantly above chance. However, no acoustic data (such as the dolphin’s echolocation signals, echoes reflected from the objects and the sample box) was present in the studies by Pack and Herman’s group, making it hard to examine what information was carried by the acoustic cues from the echoes. Furthermore, in Pack and Herman’s experiments, the PVC pipes were all filled with dry sand to achieve negative buoyancy for immersion in water and to reduce the internal reflections. Little is known about whether the accuracy of the successive match would be influenced when the material composition of the objects is changed (e.g., filling different materials inside the pipes or using pipes made by different materials). Therefore, to further investigate how the shape information might be accessible to both the dolphins’ visual and echolocation senses, it is important for us to gain an understanding of what is the nature of the echoes the dolphins receive from the interrogated objects, how do they utilize the features from these echoes that provide them information about the shape, as well as whether different material compositions would interfere with their cross-modal matching ability.

In this study, we conducted E–V cross-modal matching experiments by only varying the material composition of the objects (while the shape, size, and position of the objects remained the same). The dolphin’s matching accuracy was found to be significantly different when he had to discriminate the objects constructed from four different types of material compositions. The dolphin was allowed to choose his position freely in the underwater object inspection in the previous training, therefore in this study, we did not restrict his movement during the underwater inspection, otherwise, we would not know the different matching accuracy was caused by the restriction of his movement or by the changes of the objects’ material composition. Due to the slightly varied position in each trial, it was extremely difficult to determine the detailed acoustic scatter field received by the dolphin experimentally to gain insight into the experiments, a finite element analysis (FEA) was performed to provide the visualization of the underlying physical mechanisms involved in the processes of click emission and reception for cross-modal target discrimination.

Finite element (FE) modelling is a well-established technique that has recently become an important complementary tool for traditional experimental methods in studying the mechanism involved in sound propagation and reception in a number of odontocete species^12–16^. The technique has been validated by finding the agreement between the simulation results and the acoustic measurement results collected on several live odontocete species^16–18^. As approximations, the models have significant advantages to provide a non-invasive way for us to study the complex internal acoustic processes resulting from the interaction of the animals and the surrounding media. Based on these numerical models, we have gained a deeper and more accurate understanding of the roles of the various forehead structures in the biosonar beam formation of some odontocete species^13,15,17–19^. With the models at hand, we can attempt to estimate the sound propagation and reception for those species which are inaccessible or untrainable for experimental work, such as for neonate animals^20^. The models also enable us to obtain the physical field information in both high temporal and spatial resolution to predict the physical processes of acoustic interaction between the animal and the detecting targets^21,22^. This type of work is hard if not impossible to investigate in experimental measurements. In this study, two-dimensional (2D) FE models were constructed to simulate the acoustic processes of a single echolocation click emission from the phonic lips in the head of an echolocating bottlenose dolphin, propagation through the complex internal forehead structures, transmission via the surrounding seawater to the targets, reflection at the target, and transmission back to the animal. The modelling data presented in this study provided insights into the reasons why the dolphin exhibited significant differences in performances when discriminating different targets in the previous experiments.

## Results

### Experiment results

**Figure 1:**
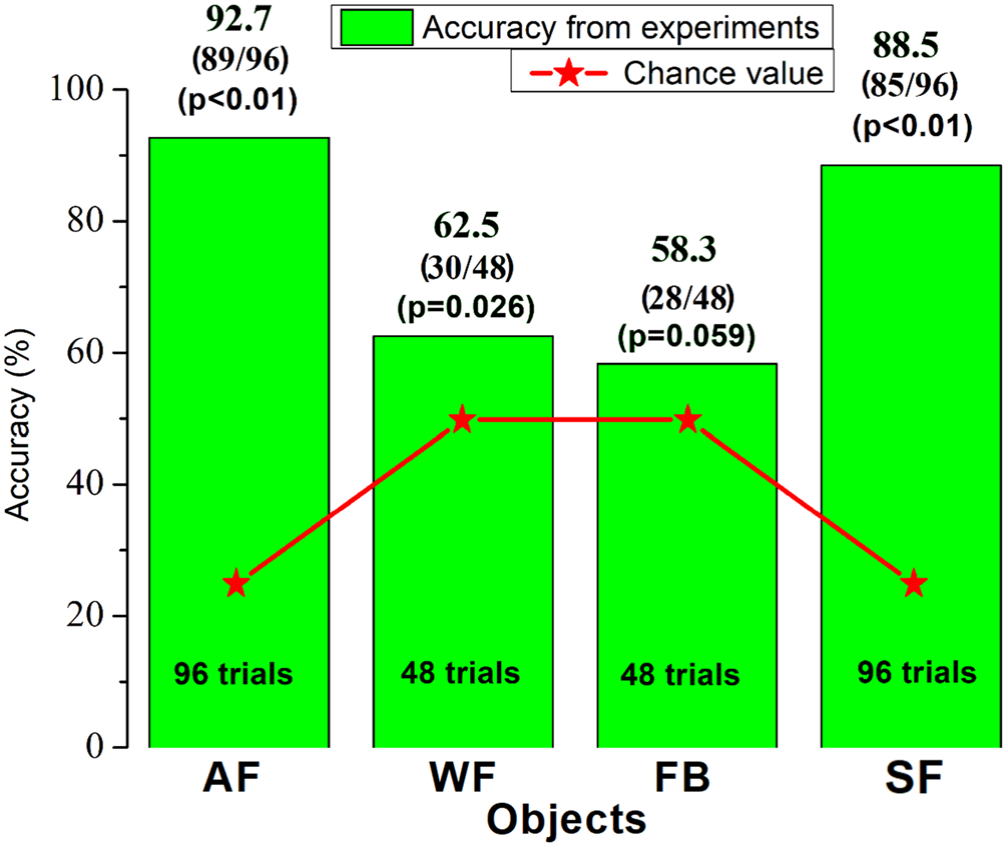
The results of E–V cross-modal matching experiments performed by the dolphin Ginsan. AF represents the air-filled PVC pipes, WF represents the water-filled PVC pipes, FB represents the foam ball arrays, and SF represents the soft closed-cell foam-wrapped PVC pipes. The experiment data for each case is provided in Supplementary Table S1.

An adult male bottlenose dolphin named Ginsan was trained to perform E-V cross-modal matching. Figure 1 shows the results from the experiments with Ginsan in discriminating four types of objects where the overall shape of the object remained consistent and only the material used to create the shapes varied: air-filled PVC pipes (AF); water-filled PVC pipes (WF); foam ball arrays (FB); soft closed-cell foam-wrapped PVC pipes (SF). The red lines represent chance values (25% for matching with four alternatives and 50% for matching with two alternatives). Binomial tests verified that the animal performed well above chance when the targets were air-filled PVC pipes (p < 0.01, matching accuracy at 92.7%). However, when the air-filled PVC objects were replaced by the water-filled PVC objects, the matching accuracy dropped off significantly (62.5% from 2 objects, p = 0.026). When foam ball arrays reconstructing the same shapes and placed in the same positions were used, the dolphin’s performance was not significantly better than chance (p = 0.059, pure matching accuracy at 58.3% from 2 objects). However, when the soft closed-cell foam-wrapped PVC pipes were used as targets, the dolphin’s performance was again significantly better than chance (p < 0.01) and the matching accuracy also rose (88.5% from 4 objects) as compared to the FB and WF cases.

### Simulation results

**Figure 2:**
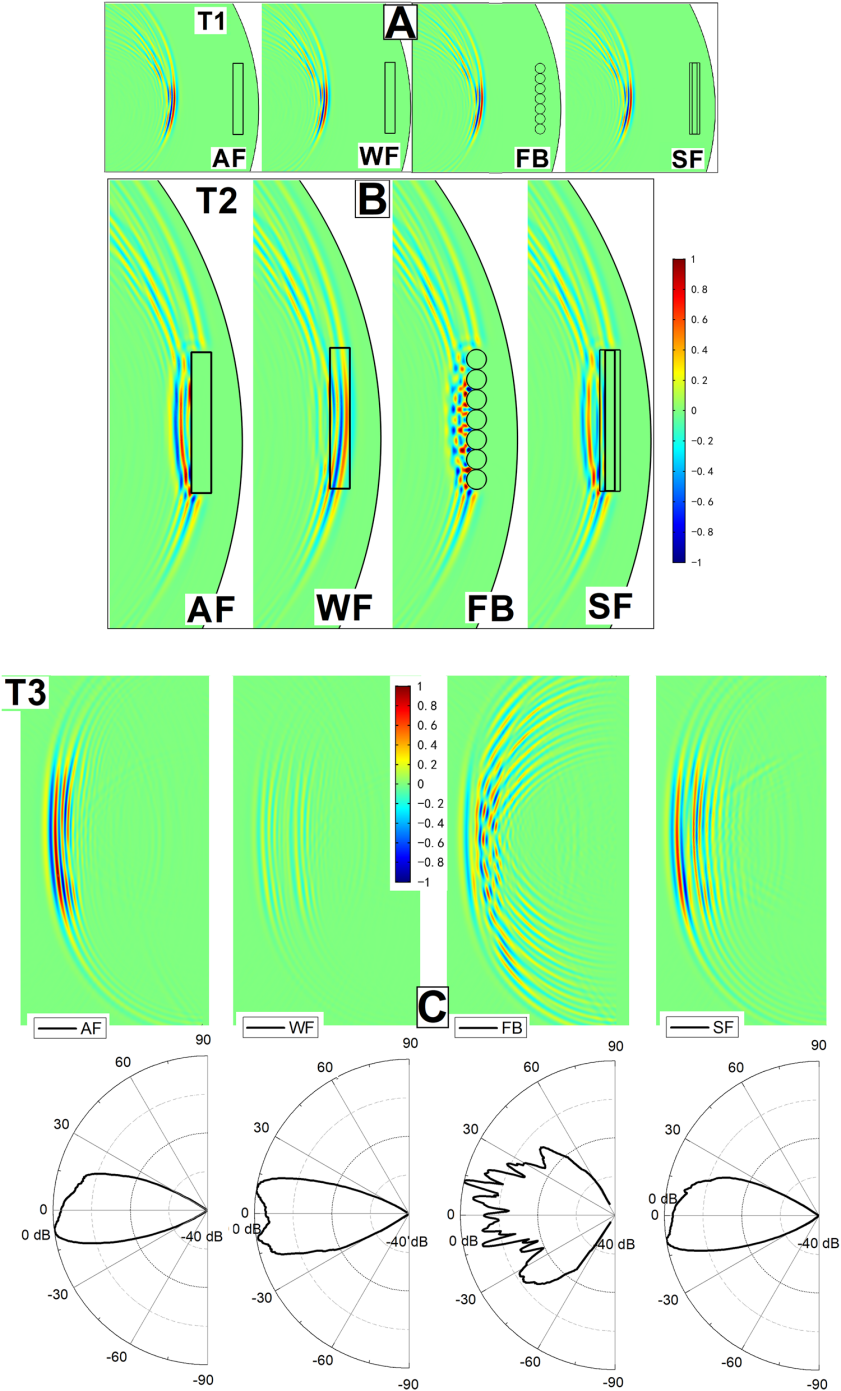
The time history of the click wavefront interacting with the object in each case. Colour bars display the relative sound pressure values which were normalized according to the maximum sound pressure value of the AF case. (**A**) T1 shows the instant where the clicks were travelling through seawater before interacting with the objects. (**B**) T2 shows the instant when the clicks were reflected and refracted by the four objects. (**C**) T3 shows the instant when the echoes were formed by the objects, transmitting back to the dolphin at a distance of 0.5 m from the dolphin’s sound source in the four cases. The corresponding beam pattern of the echo in each case is plotted in the bottom panel. The detailed acoustic processes from T2 to T3 for each case are provided in Supplementary Video S1.

To understand the backscatter mechanisms involved in the discrimination task for different type of objects, a 2D FE vertical model was used to examine the acoustic reflections from simple cylindrical targets composed of different materials. A time history of the click wavefront travelling through the dolphin’s head and the outside media was determined for each case. The wavefront at three instances (T1, T2, T3) were captured from the data and are shown in Fig. 2. They depict the changes in the wavefront as the clicks travelled through the four objects and the echoes were formed.

T1 shows the instant that an echolocation click being emitted from the animal’s head and propagating towards the objects. The wavefront at the moment before the signals hit the objects in all the cases were exactly the same. The directivity index (DI) and 3-dB beamwidth of the simulated beam in the far-field was 24.4 dB and 11.1°, respectively. T2 shows the instant the click arrived at the interfaces of the objects with the adjacent media. In the AF case, due to the large acoustic impedance mismatch between water and air, the click was completely reflected by the air inside the PVC pipe. In the WF case, the click travelled through the PVC pipe and most of the click energy was transmitted through the PVC pipe, whereas very little energy was reflected by the back face (away from the dolphin) of the PVC pipe. Overall, this resulted in a weak echo (see Fig. 2C) returning to the dolphin in the WF case. The ratio between echo level and emitted click level at 0.5 m from the dolphin’s sound source was only − 23.1 dB in this case. In the FB case, a clear pattern of interference was observed due to both the acoustic properties and the shape of the foam balls. The sound waves were reflected and scattered in all directions by the FB structure. In the SF case, some of the sound wave energy was reflected by the outer closed-cell foam material (water-closed-cell foam interface), while the remaining portion of the click went through the closed-cell foam material and was reflected by the air inside the PVC pipe. T3 shows the instant in which the echoes were transmitted back to the animal in each case. In the AF case, there was an echo (− 9.1 dB between echo level and emitted click level at 0.5 m from the dolphin’s sound source) formed mostly due to reflection at the PVC-air interface (due to presence of air inside the PVC pipe). The returning echo had only one main beam with a 3-dB beamwidth of approximately 16.2°. In the WF case, the echo was reflected as it travelled through two layers of the PVC pipes, resulting in the main beam with a 3-dB beamwidth of approximately 14.4°. The intensity of the echo was low (− 23.1 dB) since most of the energy was radiated into the water when the sound waves travelled through the pipe. In the FB case, multiple sidelobes were clearly observed in the beam pattern of the resultant echo as each part of the object scattered and reflected sound energy into different directions. In the SF case, the echo was significantly stronger than that of the WF case (− 10.2 dB between echo level and emitted click level at 0.5 m from the dolphin’s sound source) and had a beam pattern very close to that of the AF case, albeit with a slightly larger echo duration (118.4 μs) than the echo in the AF case (77.6 μs), suggesting a close resemblance to the AF case.

**Figure 3:**
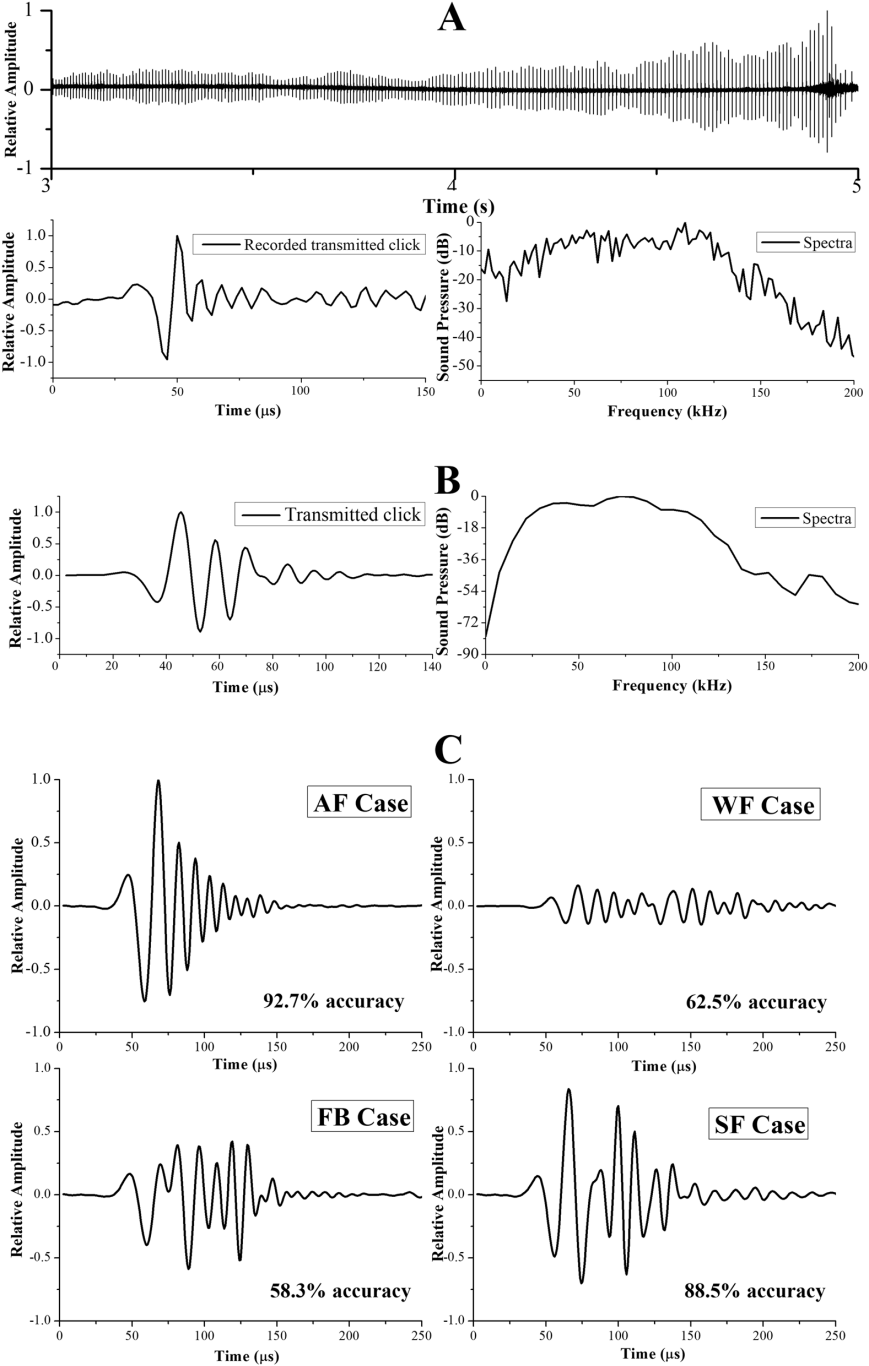

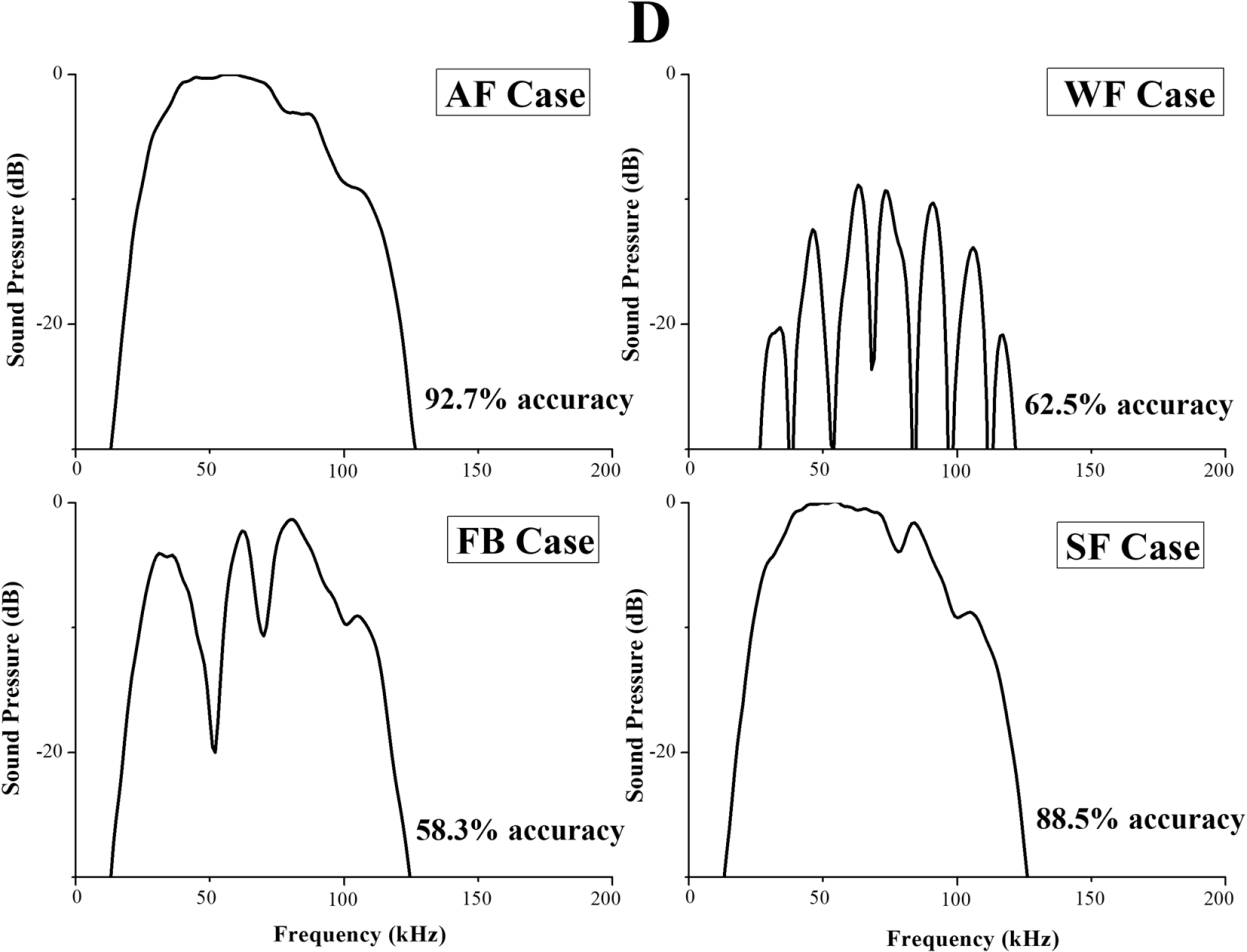
The recorded signals and simulated signals. (**A**) The waveform and corresponding spectra of a single transmitted click from a click train recorded in the AF trial. (**B**) The waveform and corresponding transmitted click at the instant of T1 from the model. Comparisons of simulated receiving echo waveforms (**C**) and frequency spectra (**D**) of four cases. The simulated signal data for each case is provided in Supplementary Table S2.

The peak-to-peak amplitudes of the transmitted clicks were normalized according to the highest peak-to-peak amplitude to obtain relative amplitudes. Figure 3A shows one of the transmitted clicks recorded in the AF case with a centroid frequency as approximately 80 kHz. Note that Fig. 3A only shows an example of the recorded transmitted signal emitted by Ginsan in one of the AF trials, characteristics of the clicks emitted by Ginsan varied slightly from trial to trial. Figure 3B displays the waveform and corresponding spectra of the transmitted click at the instant of T1 in Fig. 2A. The centroid frequency of the click is approximately 68 kHz. The waveform and frequency spectra of echoes received by the dolphin closest to the lower jaw in four cases were compared, as shown in Fig. 3C,D. The amplitude of the waveforms was relative to the highest absolute amplitude in the AF case. The amplitude of the spectra was relative to the highest absolute amplitude (0 dB) in the AF case. The overall amplitudes of the WF and FB cases were significantly lower than that of the AF and SF cases. The waveform of the SF case had two clear highlight peaks, separated by ~ 30 μs (Fig. 3C). The first peak was formed due to the reflection when the click reached the water-closed-cell foam interface. The rest of energy was transmitted through the closed-cell foam, it then reached closed-cell foam-PVC interface and PVC-air interface, creating the second peak. Figure 3D shows that the frequency spectra of the receiving echoes in the AF and SF cases were close, most energy were in the range between 40–125 kHz. The two highlight peaks in the waveform of SF case appeared in the spectra as an interference effect at frequencies around 70 to 80 kHz. By contrast, some interference patterns can be clearly observed in the spectra of the WF and FB cases. In the WF case, a small amount of the incident signals was reflected by the two boundaries of the PVC pipes, showing a distinct multipath effect. In the FB case, due to the geometry of the foam balls, the boundary of each foam ball reflected the incident signals and caused superposition in the echoes. To quantify the feature of the waveforms, we calculated the energy flux density, duration, centroid frequency, RMS bandwidth, and 3-dB beamwidth of the simulated receiving echo (Fig. 3C,D) for each case. The comparison is shown in Table 1.

**Table 1:** Energy flux density, duration, centroid frequency, RMS bandwidth, and 3-dB beamwidth of the simulated receiving echoes in each case, as well as the five parameters of the emitted click (EC) at 1 m away from the phonic lips in the model.

	Energy flux density (dB re 1 μPa^2^ s)	Duration (μs)	Centroid frequency (kHz)	RMS bandwidth (kHz)	3-dB beamwidth (degree)
AF	170.0	77.6	61.2	20.4	16.2
WF	144.6	206.4	74.8	20.0	14.4
FB	164.6	114.4	67.4	24.0	1.9
SF	170.0	118.4	61.5	20.5	18
EC	174.3	65.6	59.9	19.8	11.1

When receiving echoes, the dolphin’s ears act as an energy detector by integrating sound energy over a short time window (approximately 264 μs)^23^. To obtain the energy within this short time window, we calculated the energy flux density of the receiving echoes for the four cases, as shown in Table 1. In addition, to make our simulation results comparable to most previous studies in odontocetes, the echo duration was estimated as − 10 dB criterion relative to the amplitude envelope^24^. From Table 1, compared to the AF and SF cases where the dolphin had better matching performances, the echoes in the WF and FB cases (where the dolphin had lower matching accuracies) had higher centroid frequencies but lower energy flux densities. The echo in the WF case had the lowest energy flux density (144.6 dB re 1 μPa^2^ s) among all cases, while the energy flux densities of the echoes in the AF and SF cases were essentially the same (~ 170.0 dB re 1 μPa^2^ s). The durations of the AF (77.6 μs) and FB (114.4 μs) cases were shorter than those of the WF (206.4 μs) and SF (118.4 μs) cases since there was only one major reflection from the air in the PVC pipe (AF) and the interface of foam balls (FB). The two main reflections created superposition in the receiving echoes of the WF and SF cases, resulting in longer durations. The centroid frequency and RMS bandwidth of the echoes in the AF case were close to those in the SF case.

## Discussion

The FE modelling technique used in this study suggests a way to account for the difference in the dolphin’s ability (Fig. 1) to match objects with different material compositions in the E-V cross-modal matching experiments. The dolphin was able to correctly perform the E-V matching when the received echoes had higher intensity (Table 1) and less sidelobes (Fig. 2C), such as the ones in the AF and SF cases. Additionally, the AF and SF cases indicate that the sound waves were mostly reflected at the interfaces between two media where a high acoustic impedance mismatch was present, such as the PVC-air interface and water-closed-cell foam interface, then directly reflected back to the animal. The thickness of the PVC pipe is only 3 mm, plus the acoustic impedance mismatch of water vs. PVC is significantly lower than that of PVC vs. air, therefore the reflection at the water-PVC interface is relatively limited in the AF case. The sound field received by the dolphin after reflection off the object, arises due to the combined effect of the backscatter from the length of the pipes rather than due to a specular reflection from a single point alone. When the object was filled with water (WF case), most of the energy of the incident sound waves was transmitted through the object due to better impedance match, therefore less was reflected (approximately 10 dB re 1 µPa lower than the AF case in Fig. 3D), and the reflecting sound waves were mainly coming from two water-PVC interfaces, probably causing the dolphin to not be able to match water-filled objects in an E-V cross-modal matching-to-sample setup. In the FB case, a complex acoustic scattered field was formed due to the acoustic properties and the shape of the foam balls. This then in turn means that spurious aliases would probably appear to the dolphin making the detection of the “real” reflective points at least very difficult if not impossible. This would suggest an explanation of the dolphin’s inability to match the foam ball version of the objects. Therefore, the complex acoustic scattered field formed by weak echoes with multiple reflections could interfere with the dolphin’s ability to perceive the shape. The animal probably was not able to fully interpret the information and may not have been able to match the shape between his echolocation sense and his visual sense. In other words, the dolphin could not find the relationship between what he “saw” in the water via its echolocation sense and what he saw via visual sense in the air.

Regarding the cross-modal experiments in our research, we have trained two bottlenose dolphins (Ginsan and Angelo) for the cross-modal matching task, but most of the data were collected from Ginsan due to limited research availability to Angelo. Both of them were able to perform cross-modal matching using the air-filled PVC targets with high matching accuracy, but only Ginsan was trained for matching using targets with different material compositions. High-frequency hearing loss may be common in older dolphins^25^ but Ginsan was about 10 years old at the time. He would also have shifted his centre frequency in sound production if he had high-frequency hearing loss^26^. However, the peak frequencies produced by Ginsan from most of our recording data were between 50–130 kHz. Therefore, Ginsan was unlikely to have high-frequency hearing loss and it was not a potential factor causing him to display a low matching accuracy in discriminating the WF and FB objects. Complex psychophysiological studies such as cross-modal matching-to-sample are often limited to the involvement of a single subject due to its training cost. Given the complexity of the task the learning process could take years depending on the subject’s training experience, research experience, etc. Training and testing more individuals will help us get a deeper understanding of dolphin cross-modal matching ablility. However, the point of this study was to show the capability of the dolphin in cross-modal matching, not to investigate the average performance of dolphins. This would only be feasible with a large number of dolphins available for training and testing, that was not the case here (and rarely is at any facility).

For most dolphin species, the beam pattern of the projected biosonar beam is significantly narrower than their vision. For example, the 3-dB beamwidth of the bottlenose dolphins is around 10°^3^. It would not allow the dolphins to have an entire target inspection within a short distance, the dolphins may have to scan sideways and up and down to build up the complete shape information. From the videos we recorded in the experiments, the top view camera captured that the dolphin slightly and quickly scanned sideways in front of the sample box in some trials. In addition, a bottlenose dolphin is able to steer its biosonar beam up to 18 degrees in the horizontal plane by altering the forehead shape^27^. Therefore, the head movement and beam steering ability could have helped Ginsan increase the scanning range to get more information about the object. However, from the side view cameras, we did not observe significant up and down head movement when the dolphins were scanning in the experiments. The potential reason could be the experiment setup, specifically the scanning position of the dolphin in the water. During the training for E-V cross modal experiments, Ginsan was allowed to choose any position in front of the sample box to echolocate on the sample object. We observed that most of the time he chose to stay at the bottom left side of the sample box during the underwater inspection. The reason is most likely related to dolphin’s adaptation to the experiment setup. The sample box was fixed at one side of the pool and a thin piece of opaque plexiglass was mounted between the animal and the sample object. With this projection angle (from the bottom left side of the box), Ginsan could effectively reduce the noise reflected from the plexiglass and the wall of the pool behind the sample box, as well as increase the scanning area.

We used a piece of opaque plexiglass instead of using eyecups to block the dolphin’s vision in the experiments. This was done to avoid the dolphin seeing the object when the eyecups accidentally drop under water and especially to eliminate the potential external interference caused by taking off the eyecups between the echolocation inspection and visual matching. With respect to the different numbers of alternative objects used in the experiments, we used a set of four alternative objects for the WF and FB cases in the initial trials, the same as for the AF and SF cases. When two alternative objects were used for Ginsan, he could match the sample based on “If it is not this one then it must be the other one” strategy. This strategy would not work if the number of the alternative objects was more than two. However, the matching accuracies of the WF and FB cases were both very low when either two or four alternative objects were used. In addition, the number of trials of the AF and SF cases (n = 96) is higher than that of the WF and FB cases (n = 48). Ginsan was at the testing facility only for limited time at Ocean Park, plus the major task of our major project was to study how the dolphins successfully performing cross-modal matching, therefore, we spent most of our efforts on the E-V and V-E tasks using “air-filled” objects. During his years of working with us, Ginsan’s cross-modal matching ability was tested under multiple conditions focusing on his cognitive processes. These experiments were carried out independently, i.e., the water filled targets were tested in a course of a different sub-project than the foam targets. While the projects were planned with a consideration of the overall understanding of the underlying mechanism of the dolphin biosonar, each experiment were designed to each task separately, which explains the overall difference in the use of the four targets.

**Figure 4:**
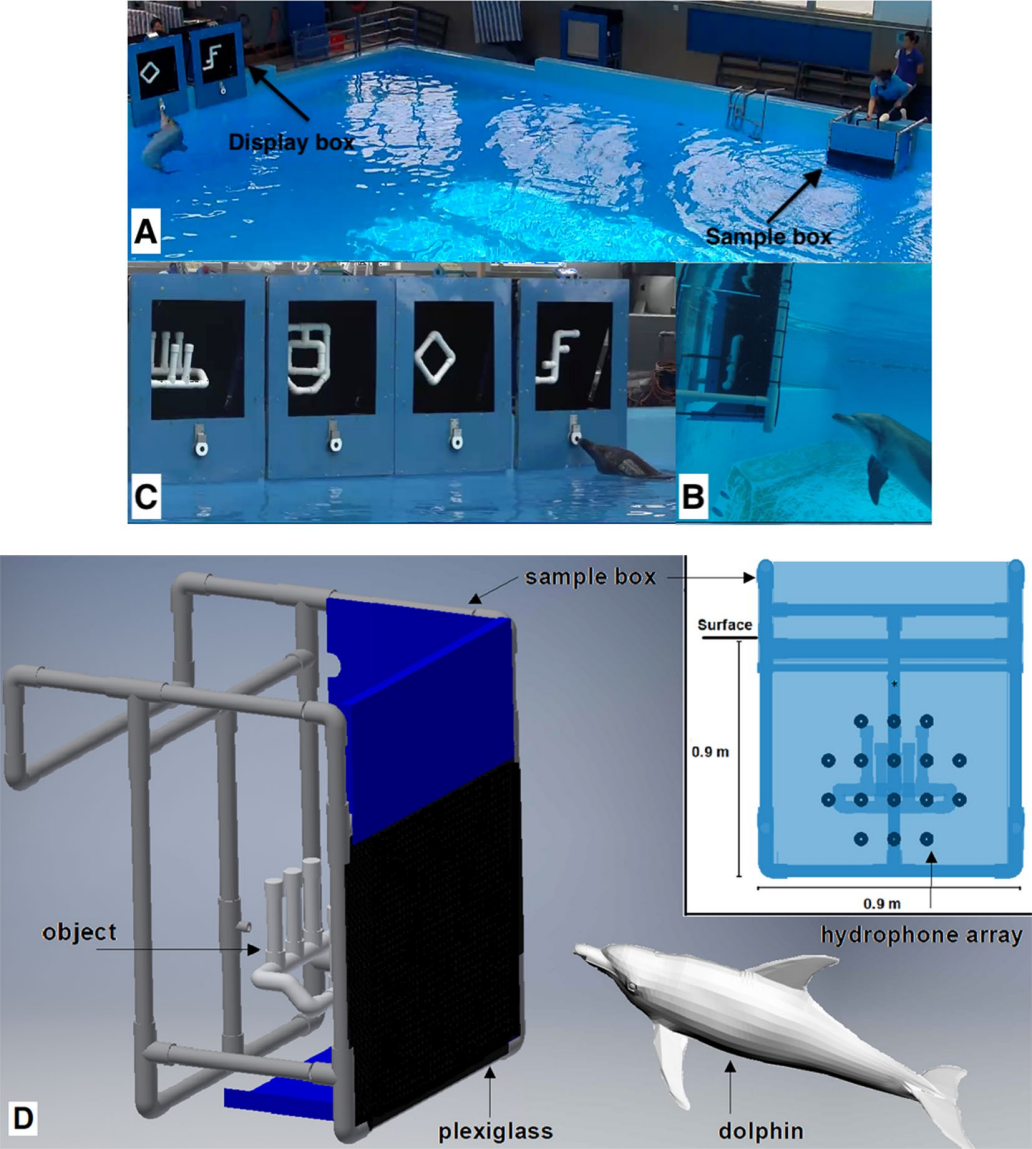
The echoic-to-visual cross-modal matching-to-sample experiment setup. (**A**) The positions of the sample box and display boxes. In the experiments, the resident dolphin used echolocation sense alone to inspect the sample object in the water on the right side of the pool, then swam up to the air and only used visual sense to match the sample on the left side of the pool. (**B**) Underwater echolocation inspection. The dolphin was echolocating on the object in front of the sample box under the water. (**C**) Visual matching in air. The dolphin was selecting the alternative object. (**D**) A 3D illustration of the experiment setup under the water. An acoustically transparent thin plexiglass (indicated in black colour in the 3D figure) was mounted on the front side of the sample box to ensure that dolphin cannot see the object inside the box. A planar hydrophone array with 16 hydrophones was placed right behind the plexiglass. A sample object was concealed in the sample box. The distance from the sample object to the dolphin was about 1 m. The recorded experiment processes are provided in Supplementary Video S2.

The four cases were conducted in the different periods of our project (see Table S1). The recording setups varied during the years, for instance, we started with using a single hydrophone on the front side of the sample box and the number of hydrophones had been increasing to 16 during the years, as well as the biteplate hydrophone was only used in several testing trials. Therefore, it is difficult to directly use the recording data from the experiment to acoustically localize the dolphin or compare the differences in the signals among cases. Furthermore, due to the fact that the dolphin’s inspection position was not fixed, the angles and distances of the echoes reflected by both the sample object and the frame of the sample box changed from trial to trial, making it not straightforward to directly infer the hydrophone recorded signals as the dolphin's perspective of the scattered field. A separate study is ongoing investigating the beamforming using recorded signals (only using data from the AF trials). This paper only focuses on using numerical models to study the backscattered field caused by the interaction between the dolphin clicks and the targets. Collecting more acoustic data for other three cases will be an important future work to further study this topic. In addition, the major objective of our research was shape discrimination through echolocation. The objects we used all consisted of complex shapes and were not limited to a sphere or single cylinder, therefore, target strength did not provide all the information required to differentiate various shapes. The dolphin’s biosonar beam is highly directional^16,28^ and Ginsan was allowed to choose the inspection location freely in the trials, thus the target strength varied across different angles and distances of signal emission. Moreover, our object shape were designed to have equal reflective surface area with the intention of having pairs of objects with similar target strength for the dolphin to discriminate. For example, the left two objects in Fig. 4C were designed to have a similar target strength while the other two objects in the right were designed as another pair. This was implemented to deter the dolphin from using only target strength to discriminate between objects and required the dolphin to have more information regarding the actual shape in order to distinguish the different objects.

It should be noted that the four 2D cylindrical shapes used in the simulations only represent the simple version of the actual objects used in the cross-modal experiment. The reflection from the actual objects in the experiment would certainly be more complex because features of the shape extend in the third dimension and echo returns from different locations on the object could return to the dolphin at the same point in time. Additionally, the models focus only on the target discrimination process in the vertical plane. However, despite the limitations, it is noteworthy that the 2D simulations presented in this study can provide crucial information with high temporal and spatial resolution to illustrate fundamental acoustic behaviours. Even though this study used currently available limited computational resources, it still leads to a better understanding of why the dolphins perceive differently when they discriminate targets with different material compositions. Scaling this analysis into a three-dimensional (3D) model would incur a massive computational cost, and it was not feasible with the available resources. However, optimizing our models to perform a 3D analysis is a very important work to be considered in the future, it would provide a more complete understanding of the entire acoustic processes and obtain a closer representation of the information dolphin receives when echolocating on a stimulus concealed in a box.

## Methods

### E–V cross-modal matching experiments

An adult male Indo-Pacific bottlenose dolphin named Ginsan was trained to perform an echoic-to-visual cross-modal MTS experiment in this study. Ginsan was born at Ocean Park Hong Kong (OPHK) and had been involved in acoustic and cognitive studies since 2005. Ginsan was housed with 14 other dolphins in the Marine Mammal Breeding and Research Centre (MMBRC) of OPHK. The experiment was conducted in one of the six interconnected pools of MMBRC (L 17 m, W 20 m and D 4 m), as shown in Fig. 4A–C. Research sessions were conducted three times daily, coinciding with the dolphin's feeding time. The research with the dolphin was approved by the Institutional Animal Care and Use Committee (IACUC) of both the National University of Singapore and OPHK, all experiments were performed in accordance with relevant guidelines and regulations. Furthermore, the study was carried out in compliance with the ARRIVE guidelines^29^.

An object with a complex shape was used as the sample stimulus and concealed in a sample box (0.9 m × 0.9 m) under water. The box and the objects were constructed using PVC pipes and fittings. Schedule 40 pipes with a thinner wall thickness were used to construct all the objects and Schedule 80 pipes with a thicker wall were used to build the sample box. To block the dolphin’s vision of the objects, a 3 mm opaque black plexiglass sheet, which was acoustically transparent in water, was placed on the front side of the box between the sample object and dolphin. A hydrophone array with 16 Teledyne Reson TC-4013 hydrophones was mounted directly behind the plexiglass to record the acoustic signals (the black dots in Fig. 4D). The TC-4013 hydrophone is an omnidirectional hydrophone with a frequency range from 1 Hz ~ 170 kHz, and the receiving sensitivity is − 211 dB ± 3 dB re 1 V/µPa. The recorded signals were also used to identify whether Ginsan sent echolocation clicks or not when he was inspecting the sample object in the water. After the dolphin had inspected the sample object echoically (Fig. 4B), the alternatives (up to four) that had been mounted in display boxes prior to each trial were revealed to the visual sense of the dolphin in air (Fig. 4C). Only one alternative physically matched the sample object. The sample object and the alternative objects were changed for each trial. Four cameras including two underwater cameras were used to record the entire process of each trial. The matching accuracy was also logged and saved for further analysis.

**Figure 5:**
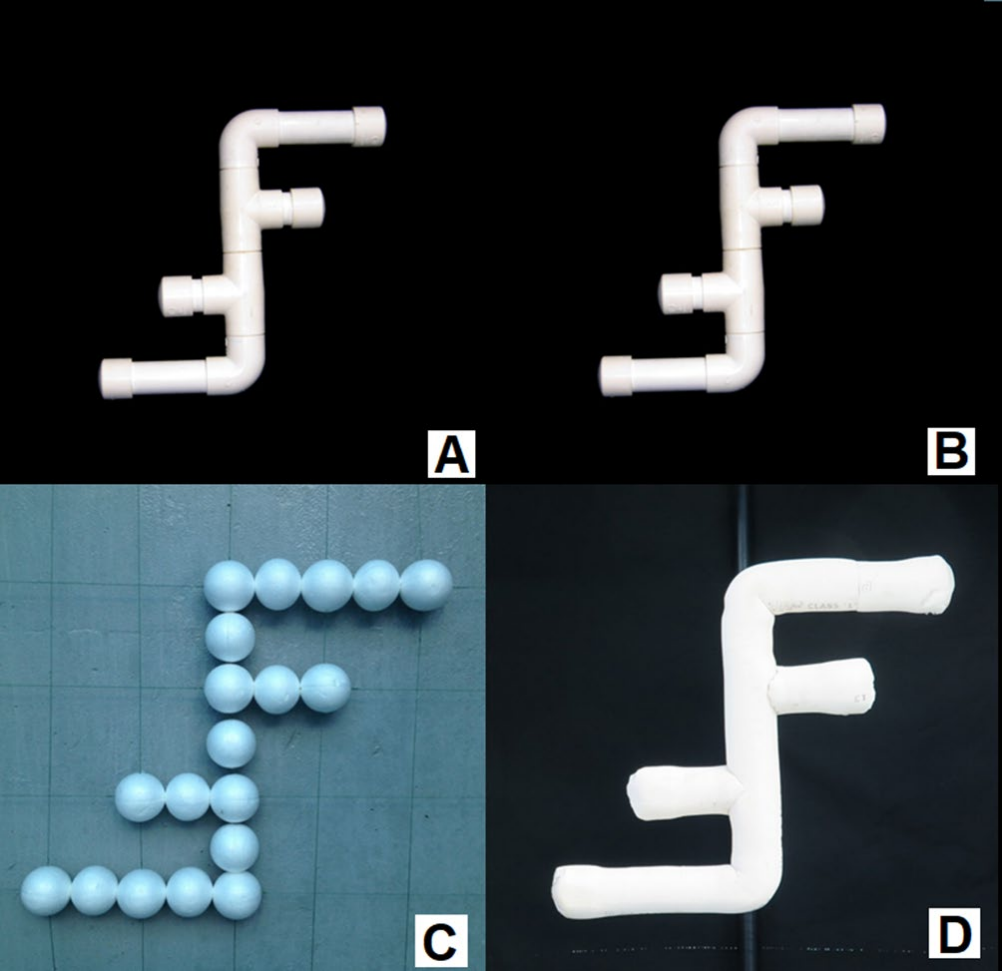
Examples of sample objects, FF-shape, with different material compositions. (**A**) The air-filled PVC pipe object. (**B**) The water-filled PVC pipe object. (**C**) The foam ball array object. (**D**) The soft closed-cell foam-wrapped PVC pipe object. The objects used in the experiments were constructed in four types of shape and made of four materials. They are all shown in Fig. 4C, from left to right: OP-shape, DL-shape, SQ-shape, and FF-shape. More details about objects’ shapes and names for each case are provided in Supplementary Figure S1.

To test the ability of the dolphin to recognize the objects constructed of different materials in the cross-modal experiments, four types of objects (air-filled PVC pipe, water-filled PVC pipe, foam ball array, soft closed-cell foam-wrapped PVC pipe) were used respectively for both sample object and alternative objects in the trials. An example of the objects using the FF-shape is shown in Fig. 5. For the air-filled PVC pipe (AF object, see Fig. 5A), the diameter of the pipes was 4.8 cm, the thickness of the pipes was around 3 mm, and the air was sealed inside the PVC pipes. For the water-filled PVC pipes (WF object, see Fig. 5B), the same PVC pipes were filled with water. The exterior appearance of the WF objects and the AF objects were exactly the same. For the foam ball arrays (FB object, see Fig. 5C), multiple foam balls of 4.8 cm diameter were arranged on 1 mm mono-filament lines to form the same overall shapes as the AF objects. For the soft closed-cell foam-wrapped PVC pipe (SF object, see Fig. 5D), an air-filled PVC pipe was wrapped by a thin layer of material (closed-cell foam). The SF objects were constructed in the same overall shape and size as the AF objects but with a smaller diameter PVC pipe (16 mm).

In the experiment, the alternative objects were also made of the different materials, so they were consistent with the sample object in each case. A binomial test was used to statistically analyse the experimental results and determine whether Ginsan’s matching performance was significant or not. A significance level of 5% was adopted for hypothesis testing (p = 0.05).

### Numerical modelling and data analysis

**Figure 6:**
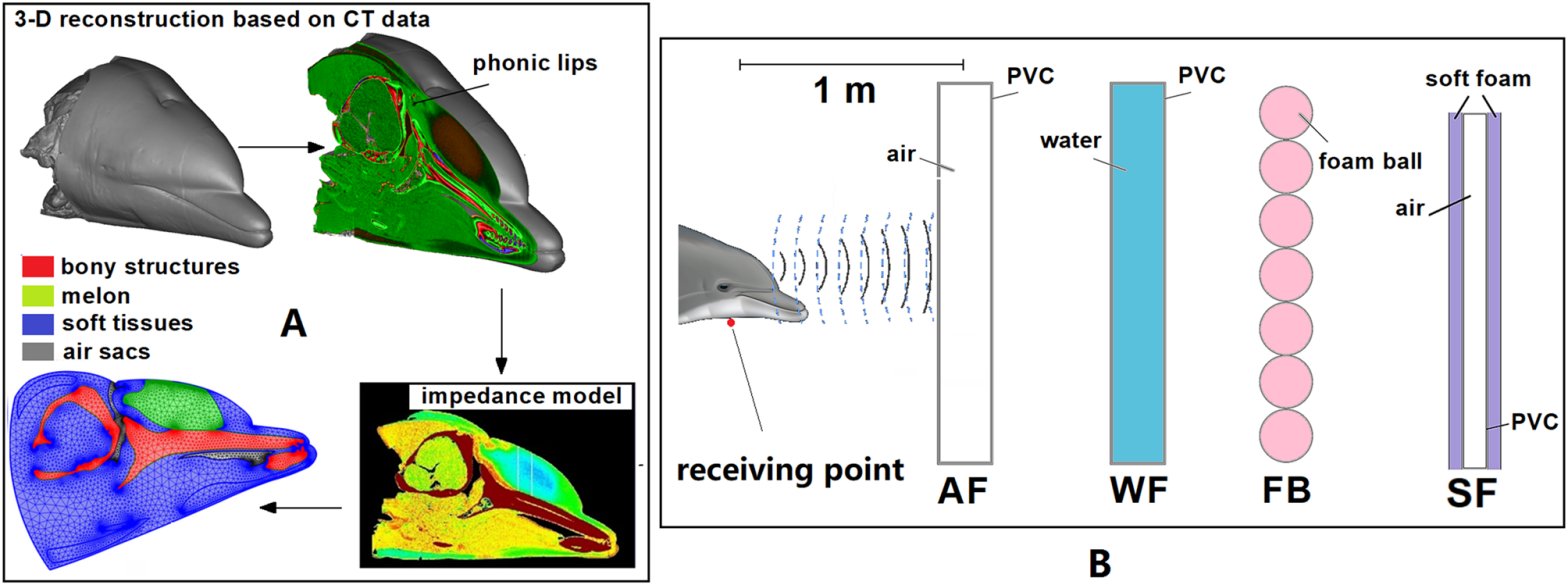
The reconstruction of four FE models. (**A**) The impedance model was reconstructed based on a slice cut through the phonic lips in the vertical plane from high-resolution CT data. The impedance model was imported into the FE software for meshing. (**B**) The FE models were used to simulate a bottlenose dolphin discriminating four 2D representation of cylindrical-shaped targets. The red dot is the receiving point close to the lower jaw of the dolphin.

The numerical model was constructed based on high-resolution computed tomography (CT) scan data of the head of a bottlenose dolphin which was provided by the Biology Department of Woods Hole Oceanographic Institution (WHOI). After reviewing the research protocol, approval from IACUC for handling and examining the cadaveric specimens was granted by the Animal Use Committee of the WHOI, all experiments were performed in accordance with relevant guidelines and regulations. The Mimics 10.1 (Materialise, Belgium) software was used for the (CT) data analysis and 3D geometrical model reconstruction, as shown in Fig. 6A. The details of the reconstruction method can be found in the previous work from Wei et al.^18^, in which CT data was combined with physical measurements of animal tissues to reconstruct an acoustic impedance model for a bottlenose dolphin’s head. A slice which was closest to the midline of the head that cut through the right side of the phonic lips was selected to create a 2D numerical model in the vertical plane, as shown in Fig. 6A.

The 2D impedance models were imported to COMSOL Multiphysics modelling software (Stockholm, Sweden) for FEA and corresponding data analysis. The simulation data was exported from the COMSOL and the figures were plotted by Origin Lab-OriginPro software (Wellesley, MA). The FE model simulated the processes of the click emission and propagation from the internal biological structures to the external media, as well as the transmitted click’s interaction with the test objects in the vertical plane. There were three main parts included in the FE models: the head of an echolocating bottlenose dolphin, the surrounding seawater, and the object. In the vertical FE model, the head of the dolphin contained the structures such as the right side of phonic lips, melon, blubber, brain, musculature, mandibular fat, connective tissue, maxilla, mandible, vestibular sac, nasal passage, and premaxillary sac. The four cylindrical shaped targets depicted in Fig. 6B were considered in the FE models, as simplified versions of the actual objects used in the MTS trials. The objects were simulated at a distance of 1.1 m from the dolphin’s sound source in each case. The sizes of the objects set in all the four cases matched those used in the experiments. For the blubber, muscle, mandibular fat, melon, connective tissue, and bony structures, the sound velocity and density values were referenced from previous work ^18^. For the media outside the dolphin’s head, the average values of salinity and temperature of the seawater measured in the pool during the experiment were 33.3 ppt and 22.8 ℃, respectively. Therefore, the sound velocity and density values of seawater in the model were set accordingly to obtain results as close as possible to the actual experiments. The sound velocity and density values of the seawater, PVC material, foam ball material, and soft closed-cell foam material were shown in Table 2^30,31^. A receiving point was added in the FE models close to the lower jaw of the dolphin^3^ in each case to monitor the simulated reflected echo from the objects, as shown in Fig. 6B.

**Table 2:** The material properties of the seawater, PVC material, foam ball material, and soft closed-cell foam material used in the simulation.

	Seawater	PVC material	Foam ball material	Soft closed-cell foam material
Sound velocity (m/s)	1527	2218	500	1700
Density (kg/m^3^)	1022	1350	300	330

In the simulation, the model was meshed into second-order triangular elements using COMSOL’s free mesher. The meshing layouts are displayed in Fig. 6A. We performed a mesh refinement analysis for the models to find the optimal element size that can obtain the solution with sufficient numeric precision. Finally, the element size for the model was set as at least ten elements per wavelength of the peak frequency of the excitation signal at source ($\uplambda ={c}_{water}/{f}_{p}$), where ${c}_{water}$ is the sound speed of the seawater (1527 m/s). ${f}_{p}$ is the peak frequency of the source signal. According to the previous measurements^3,32,33^, bottlenose dolphins emit signals with lower peak frequencies (30–60 kHz) for target detection in the tanks, therefore the peak frequency was set as 60 kHz, then the wavelength $\uplambda$ is 0.02545 m. The low-reflecting boundary condition^34^ was applied to simulate the transmission of a single echolocation click and its backscattering from the objects, without considering the effect of reverberation from the boundaries.

In the previous studies by Wei et al.^16,18^, the FEA was used to simulate a single click emitted in the head of a bottlenose dolphin which then transmitted from the near acoustic field to the far acoustic field in both the vertical and horizontal plane. The models were confirmed and validated with actual biosonar signal measurements in signal characteristics and beam patterns on and around the heads of the live bottlenose dolphins^16,18^. The current work employed the same methods by performing the transient time domain finite element computation. The time steps were set as 0.8 μs to obtain a very detailed sound transmission and reception process. Madsen et al.^35,36^ found that the right phonic lip is the site at which the echolocation clicks are generated. Consequently, the sound source was placed at the right phonic lips in the models. In the sagittal section of the CT data, the size of the right phonic lips was approximately 3–4.5 mm, which was significantly smaller than the wavelength (25.5 mm) in the model. Thus, we decided to use a point source to model the source region for the vertical FE models. A short-duration broadband pulse with an RMS bandwidth of 37.7 kHz was generated at the source location of the FE model as sound source excitation. The equation of the pulse can be written as:

1 $${Q}_{m}=-A{e}^{-2{\pi }^{2}{f}_{0}^{2}{(t-{t}_{p})}^{2}}{{\rm sin}}(2\pi {f}_{0}t)$$

2 $${t}_{p}-\frac{1}{{f}_{0}}<t<{t}_{p}+\frac{1}{{f}_{0}}$$

where $\mathrm{A}$ is the pulse amplitude (Pa), ${f}_{0}$ is the centre frequency (Hz), ${t}_{p}$ is the time from the onset of the signal to its peak amplitude (s), and $t$ is the time (s). The time of this pulse has to satisfy Eq. (2).

Since the sound pressure of the backscatter from the four objects were different, to better display the directivity in Fig. 2, the sound pressure values were normalized with respect to the highest values in each respective case. The sound pressure values were then converted into dB scale (SPL = A + 20log10P), where P is the normalized peak-to-peak sound pressure of the signal at each point, and A is a constant. The beam patterns of the returning echoes were plotted by determining the SPL of an echo reflecting from the centre of each object over a circle with a radius of 0.5 m in each case.

## Conclusions

Early work has shown that dolphins can perceive the shape of objects composed by dry sand-filled PVC pipes through echolocation and match that information through the visual sense^8–11^. However, whether changing the material composition of the objects would still provide enough information for the animal to discriminate the objects in a cross-modal matching setup had not been studied to date. To address this question, we trained a bottlenose dolphin to perform E–V cross-modal match-to-sample experiments using four types of objects differing only in material composition, but not in shape. The results showed that the dolphin was unable to match the water-filled PVC and foam ball array versions of the objects. To visualize the complex acoustic process and study the underlying physical mechanism, simplified CT based FE models were developed. The simulation results indicate that the excessive scattering of the incident acoustic field on the object, or formation of the low intensity acoustic field from multiple weak reflections, was most likely the reason for the dolphin’s inability to perceive and match the shape for these trials.

The study also speculates the possible reason for dolphins mistakenly swallowing plastic objects in the water, which could provide important information for studying ocean plastic pollution impacts on cetaceans in the future. Plastic pollution is posing threats to marine animals including cetaceans. A recent study has examined the stranded cetaceans including different species of whales, dolphins, and porpoises in Ireland^37^. Their post-mortem examinations reported that 8.5% of these animals had plastics, which are unable for them to digest or excrete, in the digestive tracts. Plastic bags and other non-degradable objects could trap or choke dolphins, especially young animals. Dolphins can generally discriminate between plastic and food, but plastics can be swallowed accidentally^38^. The WF case studied in our study may shed some light on why the dolphins could mistakenly swallow plastics (e.g., plastic bags filled with water, small plastic bottles filled with water) even they have echolocation. Dolphins are likely to use both visual and echolocation sense simultaneously, to recognize objects with a relatively higher accuracy^39^, when there is enough light in the water. Acoustic cues (e.g., amplitude, highlight structure, frequency content, etc.) play important roles in their cross-modal target discrimination. As those plastics filled with water, it may interfere with dolphin’s ability to target discrimination, especially for the younger dolphins^40^ whose acoustic structures are not fully formed. For example, the lipid components in the animal’s melons change with growth, a previous study showed that no significant gradient of sound velocity or density was found in the heads of a neonate and a one-year-old juvenile finless porpoises^41^. More evidence needs to be collected to provide conclusive results.

## Supplementary Information

Supplementary Information 1.

Supplementary Information 2.

Supplementary Information 3.

Supplementary Information 4.

Supplementary Information 5.

Supplementary Information 6.

Supplementary Information 7.

Supplementary Information 8.

Supplementary Information 9.

Supplementary Information 10.

Supplementary Information 11.

Supplementary Information 12.

Supplementary Information 13.

Supplementary Information 14.

Supplementary Information 15.

Supplementary Information 16.

Supplementary Video 1.

Supplementary Video 2.

Supplementary Video 3.

Supplementary Video 4.

Supplementary Video 5.

Supplementary Video 6.

Supplementary Video 7.

## Data Availability

All data generated or analysed during this study are included in this published article (and its Supplementary Information files).
