# Supplementary material for: Possible limitations of dolphin echolocation: a simulation study based on a cross-modal matching experiment
Source: Sci Rep. 2021 Mar 23;11:6689. doi: 10.1038/s41598-021-85063-2 (PMC7988039; doi:10.1038/s41598-021-85063-2)
Supplement: Supplementary file 14 — Supplementary Information 14. [file 41598_2021_85063_MOESM14_ESM.pdf]

Possible limitations of dolphin echolocation – a simulation study based on cross-modal matching experiment

Chong Wei[1,*], Matthias Hoffmann-Kuhnt[2], Whitlow W. L. Au[3], Abel Ho Zhong Hao[2], Eszter Matrai[4], Wen Feng[5], Darlene R. Ketten[6,7], Yu Zhang[5,8]

[1]Centre for Marine Science & Technology, Curtin University, Kent Street, Bentley WA 6102, Australia
[2]Acoustic Research Laboratory, Tropical Marine Science Institute, National University of Singapore, 18 Kent Ridge Road, Singapore 119227, Singapore
[3]Hawaii Institute of Marine Biology, University of Hawaii, 46-007 Lilipuna Road, Kaneohe, HI 96744 USA
[4] Research Department, Ocean Park Hong Kong, Hong Kong (SAR), China
[5] College of Ocean and Earth Sciences, Xiamen University, Xiamen 361005, P. R. China
[6]Department of Biomedical Engineering, Boston University, Boston, MA 02215 USA
[7]Department of Otology and Laryngology, Harvard Medical School, Biology Department, Woods Hole Oceanographic Institution, Woods Hole, MA, USA
[8]Key Laboratory of Underwater Acoustic Communication and Marine Information Technology of the Ministry of Education, Xiamen University, Zengcuoan West Road, Xiamen, 361005, P. R. China.
*corresponding author: weichong3310@foxmail.com

Case	Correct	Incorrect	Accuracy
AF	89	7	92.7%
WF	30	18	62.5%
FB	28	20	58.3%
SF	85	11	88.5%

Table S1: Echoic-Visual (E-V) Cross-modal matching experiment results from four case. In each case, the following objects were used: air-filled PVC pipes (AF); water-filled PVC pipes (WF); foam ball array (FB); foam-wrapped PVC pipes (SF). The detailed records of each trials in each case were provided in supplementary files. In the records, DL, FF, OP, SQ represent four types of objects with different shapes used in the experiments, the photos of these objects are shown in the Figure S1.

Video S1: The detailed processes when the echoes reached the objects in each case.

The videos for the four cases are provided in supplementary files.

Table S2: The simulated waveforms of the receiving echoes in each case. The data of the echoes of four cases were exported from COMSOL modelling software, the data is provided in supplementary files.

Video S2: The three videos recorded by different cameras in the experiments. The video recorded by the top view camera shows how Ginsan inspected the sample object in the box in the water and how the trainers replaced the sample object between the trials. The underwater side view camera recorded the process when Ginsan inspected the sample object in the water. In this video, an air-filled object was used as a sample object in the box. Note that the cross hydrophone array in this video was only used in several testing trials in the beginning. To avoid the potential inference on dolphin matching performances, later we decided not to use the cross hydrophone array in the rest trials. In addition, the dolphin made a relatively quick decision on all targets. There was no significant difference between his performance on the various objects. The camera in the air shows how Ginsan found the match in the air after the underwater inspection. In this video, air-filled targets were also used as alternative objects. The videos are provided in supplementary files.

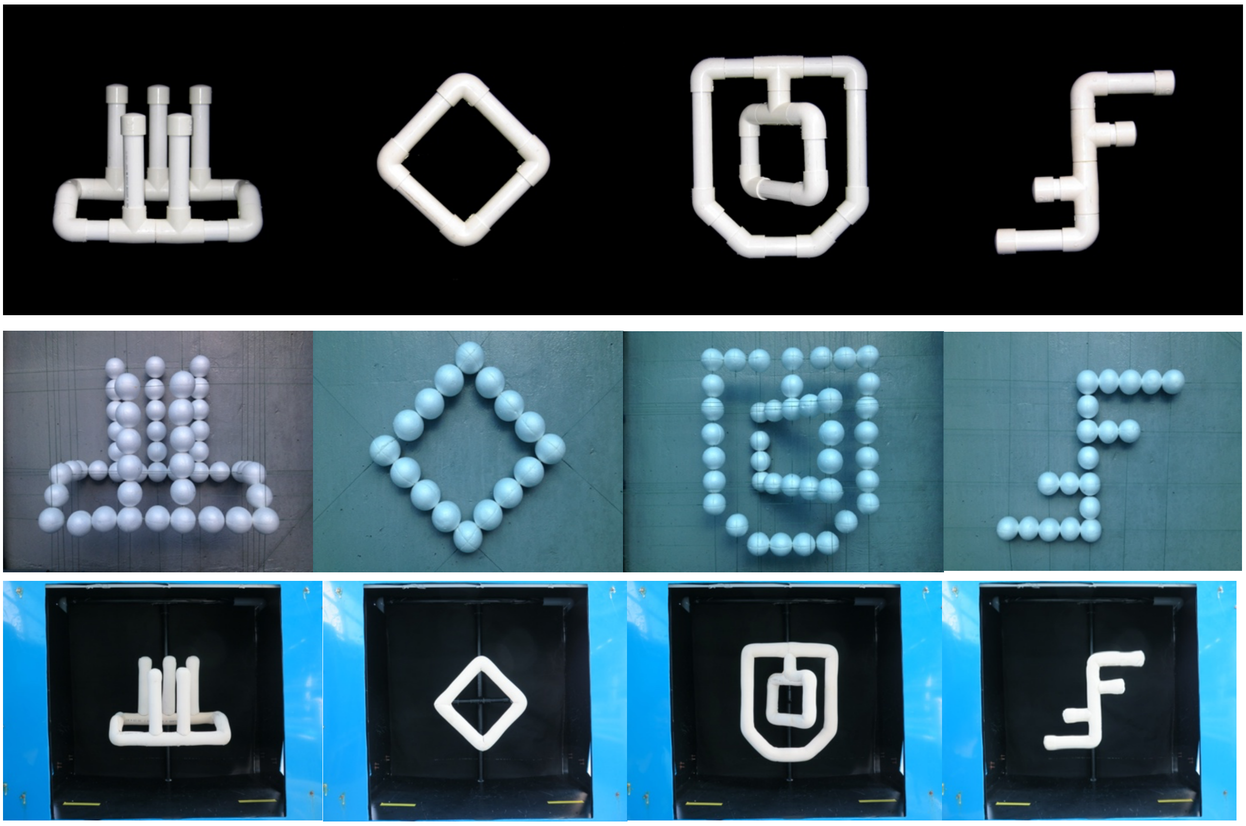

Figure S1: Four types of objects used in the cross-modal matching experiment. The objects showed in first row are the Baseline objects, which are the objects used in AF case. Note that the objects used in AF and WF cases looked exactly the same from outside, therefore, only objects used in AF case are provided in the supplementary files. The objects showed in the second row are the foam ball objects (FB case). The objects showed in the third row are the full foam objects (SF case). The objects in the first column named OP, the objects in the second column named SQ, the objects in the third column named DL, and the objects in the fourth column named FF, respectively. They represent four types of objects with different shapes in the experiment.
